# Serum Starvation Induces DRAM Expression in Liver Cancer Cells via Histone Modifications within Its Promoter Locus

**DOI:** 10.1371/journal.pone.0050502

**Published:** 2012-12-12

**Authors:** Peihua Ni, Hong Xu, Changqiang Chen, Jiayi Wang, Xiangfan Liu, Yiqun Hu, Qishi Fan, Zhaoyuan Hou, Yang Lu

**Affiliations:** 1 Department of Pharmacy, Ruijin Hospital, Shanghai Jiaotong University, School of Medicine, Shanghai, China; 2 Department of Laboratory Medicine, Ruijin Hospital, Shanghai Jiaotong University, School of Medicine, Shanghai, China; 3 Department of Biochemistry and Molecular Cell Biology, Shanghai Key Laboratory for Tumor Microenvironment and Inflammation, Shanghai Jiaotong University, School of Medicine, Shanghai, China; Baylor College of Medicine, United States of America

## Abstract

DRAM is a lysosomal membrane protein and is critical for p53-mediated autophagy and apoptosis. DRAM has a potential tumor-suppressive function and is downregulated in many human cancers. However, the regulation of DRAM expression is poorly described so far. Here, we demonstrated that serum deprivation strongly induces DRAM expression in liver cancer cells and a core DNA sequence in the DRAM promoter is essential for its responsiveness to serum deprivation. We further observed that euchromatin markers for active transcriptions represented by diacetyl-H3, tetra-acetyl-H4 and the trimethyl-H3K4 at the core promoter region of DRAM gene are apparently increased in a time-dependent manner upon serum deprivation, and concomitantly the dimethyl-H3K9, a herterochromatin marker associated with silenced genes, was time-dependently decreased. Moreover, the chromatin remodeling factor Brg-1 is enriched at the core promoter region of the DRAM gene and is required for serum deprivation induced DRAM expression. These observations lay the ground for further investigation of the DRAM gene expression.

## Introduction

Inactivation of cell-death pathways is a central component of cancer progression [1]. Various mechanisms exist in normal human cells to invoke cell death and eradicate damaged cells that may grow aberrantly to form tumors [2]–[4]. Under normal conditions macroautophagy (hereafter referred to as “autophagy”) is a tightly regulated membrane-trafficking process for degrading and recycling of cytosolic proteins and organelles at basal levels. It can be induced above the basal level in response to diverse stimuli including nutrient starvation, infection, genotoxic agents, or cytokines [5]–[8]. Autophagy may function in different contexts to either promote or inhibit cell survival [1], [5], [7], [9]–[11], suggesting that it may play an important pathological role in carcinogenesis.

Human *DRAM* (damage-regulated autophagy modulator) gene was identified as a p53-activated gene which encodes a highly conserved lysosomal membrane protein and is constituted of 238 amino acids in length [12]. DRAM is not only critical for the ability of overexpressed p53 or DNA damage-activated p53 to modulate autophagy [13], but also for p53's ability to induce apoptosis [9]. The DRAM is specifically localized on lysosomes, an organelle participating in the last step of autophagy [12]. Therefore it is plausible that DRAM may regulate the autophagosome-lysosome fusion, a process required for the generation of autophagolysosomes.

It has been shown that DRAM has a potential tumor-suppressive function and is downregulated in many human cancers [12]. The downregulation of DRAM mRNA in these cancer cells occurs both by direct hypermethylation within the CpG island in the promoter region of this gene and by other, as yet unidentified, mechanisms such as epigenetic modifications of core histones near the DRAM gene [12]. In this report, we characterized the human DRAM gene promoter and identified the DNA sequences essential for its regulation.

## Results

### Serum starvation stimulates DRAM expression in liver cancer cells

**Figure 1:**
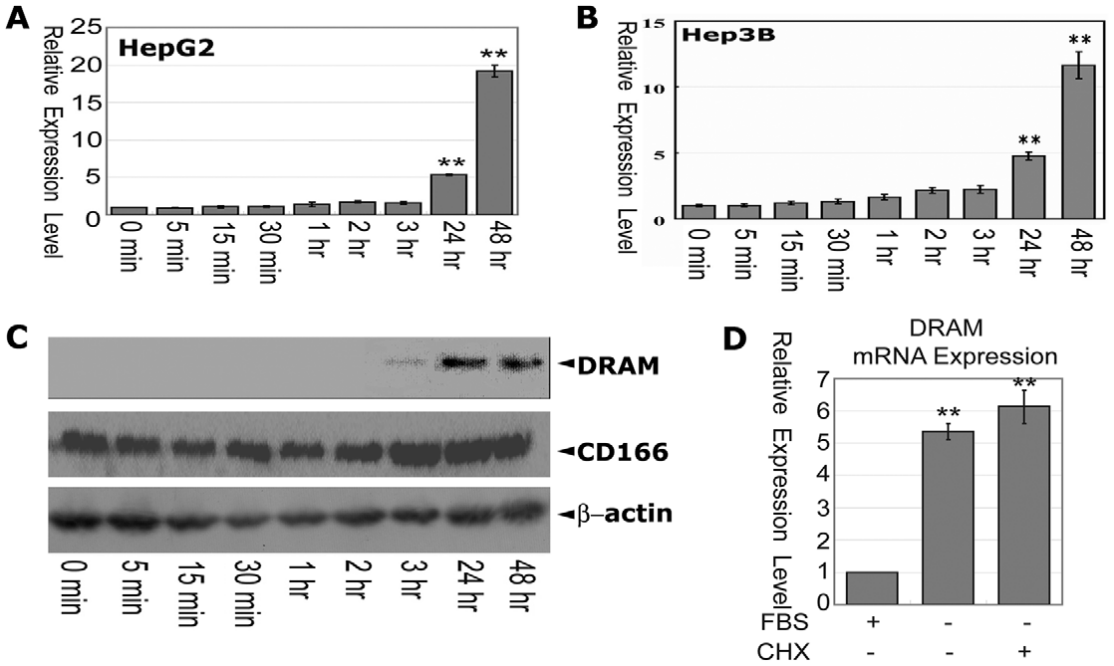
Serum deprivation induces DRAM expression in liver cancer cells. (A) mRNA level of the DRAM gene upon serum deprivation at different time points were quantified by real-time RT-PCR in HepG2 cells. (B) DRAM expression in Hep3B cells is similar to that observed in HepG2 cells. (C) Analysis of the DRAM protein expression in HepG2 cells. CD166 served as a positive control for serum starvation induced protein expression. Cells were grown to 70% confluency in DMEM containing 10% FBS. These cells were washed with PBS and fed with DMEM media omitting FBS for various time points. The resulting cells were harvested and the total RNAs and whole cell lysates were extracted and the expression of DRAM mRNA and protein was examined by qRT-PCR or western blot. GAPDH were used as an internal control. (D) Inhibition of protein synthesis does not affect serum starvation-induced DRAM expression in HepG2 cells. Cells were treated with CHX (10 mM) under serum deprivation for 24 hrs, and total RNA was extracted for analysis of the DRAM expression by real-time PCR. All results are presented as means±S.D. of three independent experiments. **, *p*<0.01 level using t-test.

Deprivation of serum induces apoptosis and autophagy in many cell types including HepG2 and HepB3 cells. These observations prompted us to examine whether serum starvation could induce DRAM expression in liver cancer cells. HepG2 and Hep3B cells were grown to 70% confluency in DMEM containing 10% FBS. These cells were washed with PBS three times and were fed with media omitting FBS for various time periods. The resulting cells were harvested and the total RNAs were extracted. The expression of DRAM mRNA was examined by quantitative RT-PCR (qRT-PCR). In both HepG2 and Hep3B cells the mRNA of DRAM was significantly induced 24 h post-serum deprivation, and reached the highest level at 48 h (Figure 1A, B) in a similar manner, suggesting that serum deprivation induces DRAM expression in liver cancer cells. The level of DRAM proteins in HepG2 cells at various time points was examined by western blot assays. Consistent to the expression of DRAM mRNA observed by qRT-PCR, DRAM protein was weakly detected at 3 hours after serum deprivation and reached high level at 24 and 48 hours of serum deprivation (Figure 1C).

To determine whether new protein synthesis was required for serum deprivation to induce DRAM expression, Cycloheximide (CHX) was applied to the serum free media for 30 minutes to inhibit new protein synthesis. Pretreatment of the HepG2 cells with CHX did not inhibit the serum deprivation induced DRAM expression (Figure 1D), suggesting that the upregulation of DRAM expression is independent of protein synthesis in HepG2 cells.

### The proximal region of the DRAM promoter is sensitive to nuclease digestion

**Figure 2:**
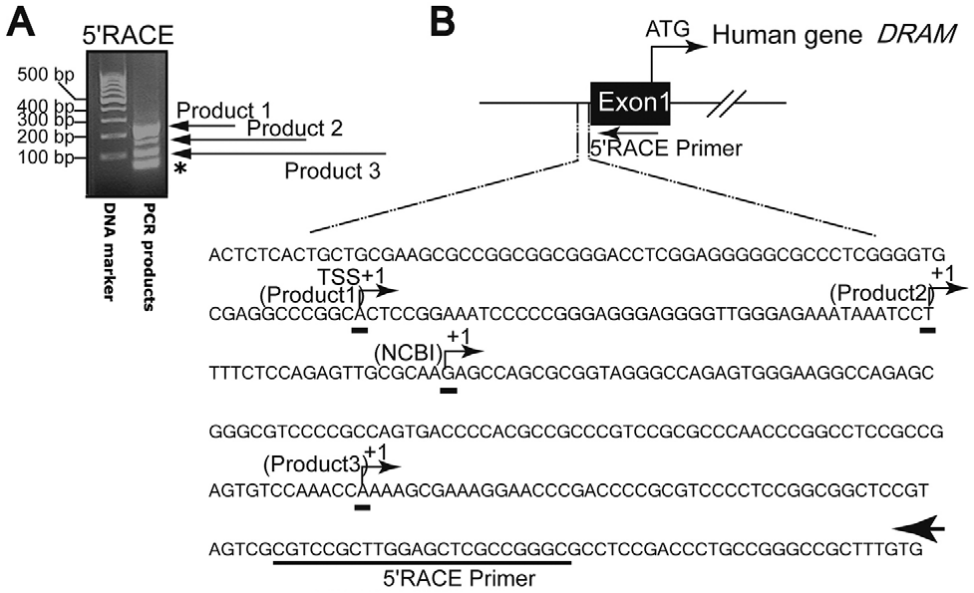
Identification of the transcription starting site by 5′RACE PCR. (A) Agarose gel showed multiple 5′RACE PCR products. (B) Mapping the potential TSS of the *DRAM gene*. The arrow indicated the nested reverse 5′RACE primer. The GeneRacer PCR, based on RNA ligase-mediated and oligo-capping rapid amplification of cDNA, was carried out according to the manufacturer's instructions. The PCR products were resolved on agarose gel, and potential bands were collected and confirmed by DNA sequencing. * indicates a non-specific amplification of the primer dimmers.

To identify the regulatory elements which response to the serum deprivation in the DRAM promoter, we first set out to determine the transcription start site (TSS) of the DRAM gene by employing 5′ RACE assays. DNA sequencing confirmed that three PCR products designated as product 1(∼216 bp), product 2 (∼172 bp), and product 3(∼106 bp) were direct amplifications from the 5′ UTR of the DRAM gene (Figure 2A). The lowest band (∼47 bp) in the gel was non-specific amplification of the primers themselves. The first nucleotides for Product 1, 2, and 3 were identified as A, T and A respectively (Figure 2B). Product 1 and product 2 were 65 bp and 20 bp upstream, while product 3 was 107 bp downstream of the first nucleotide of the DRAM cDNA sequence found in the Gene Bank of the National Center for Biotechnology Information (Gene ID: 728655). For convenient description hereafter, we arbitrarily referred the nucleotide A in the product 1 as the TSS (+1).

**Figure 3:**
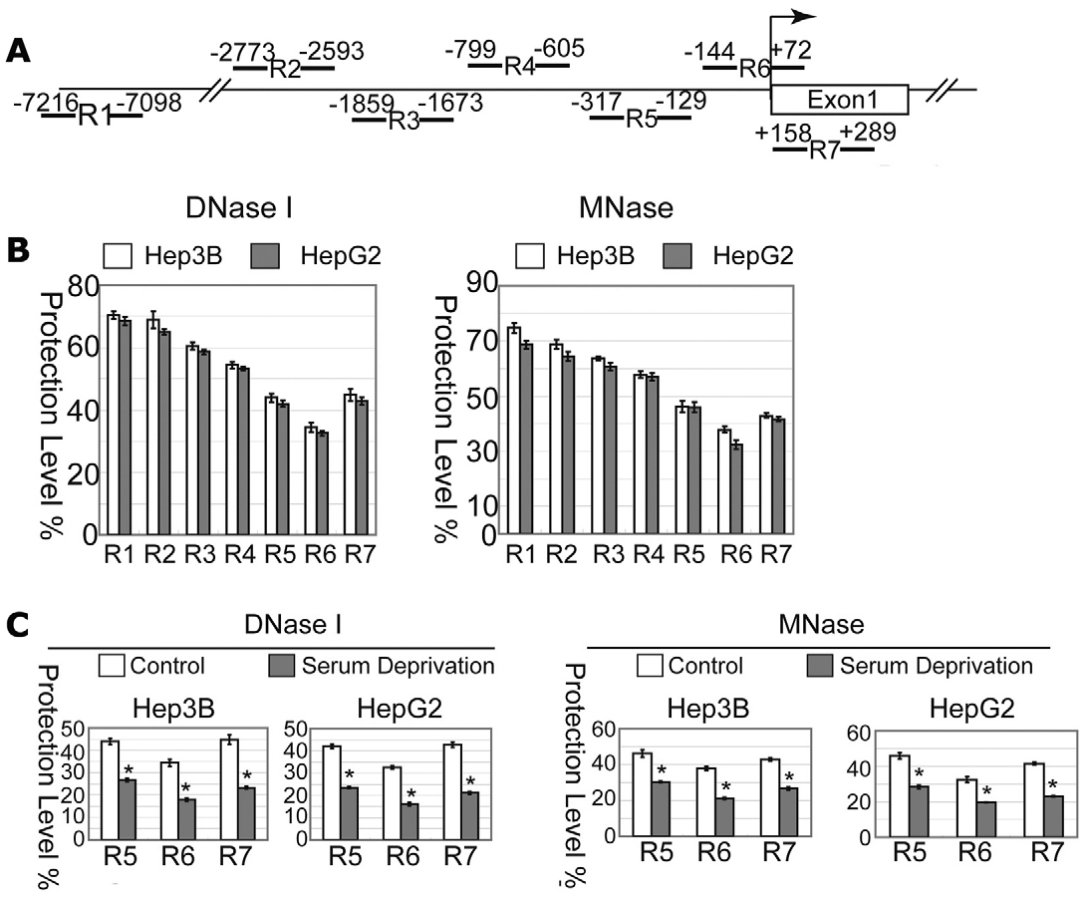
The proximal region of the DRAM promoter is sensitive to nuclease digestion. (A) Schematic representation of the primer sets used for CHART-PCR. (B) R6 region of the DRAM promoter is more sensitive to DNase I and MNase digestion. The genomic DNA was isolated from Hep3B and HepG2 cells and analyzed by quantitative PCR. The data was shown as percentage of digested DNAs to undigested DNAs. (C) Serum deprivation increases the accessibility of R5, R6 and R7 regions to nuclease digestion in Hep3B and HepG2 cells.

To further provide clues in search for the regulatory elements at the DRAM promoter, we performed CHART-PCR assays to evaluate the accessibility of the DRAM promoter DNA to nuclease digestion. Nuclei isolated from HepG2 and Hep3B cells were subjected to digestion with DNase I or MNase and the resulting genomic DNA fragments between −7216 and+289 bp (relative to the location of TSS) were analyzed using quantitative PCR assays (Figure 3A). The data were presented as the percentile between the intact DNA from the nuclease-treated samples and the untreated, and were inversely reflected the proportion of the protected DNA. The DNA fragments spanning the proximal promoter (R5, R6 and R7) were more sensitive to DNase I and MNase digestion, with particularly R6 (from −144 to +72) exhibiting the lowest protection of ∼35% in both cells examined, while higher levels of protection against DNase I or MNase digestion were observed at the R1 and the R2 region, indicating that the DNase I and MNase accessibility are limited to the proximal promoter region, especially to the R6 region (Figure 3B).

To examine the effect of serum deprivation on the DNA accessibility of the DRAM promoter to DNase I and MNase digestion, nuclei isolated from HepG2 and Hep3B cells grown in serum free media for 48 hours were subjected to nuclease digestion and CHART-PCR analysis. The accessibility of all R5, R6 and R7 regions to nuclease digestion was significantly increased in both cells upon serum deprivation with the R6 region displaying the least protection level (Figure 3C). Collectively, these data implicate that the proximal region of the DRAM promoter contains putative cis-regulatory elements responsive to serum deprivation.

### Serum starvation induces changes in histone modification at the DRAM promoter loci

**Figure 4:**
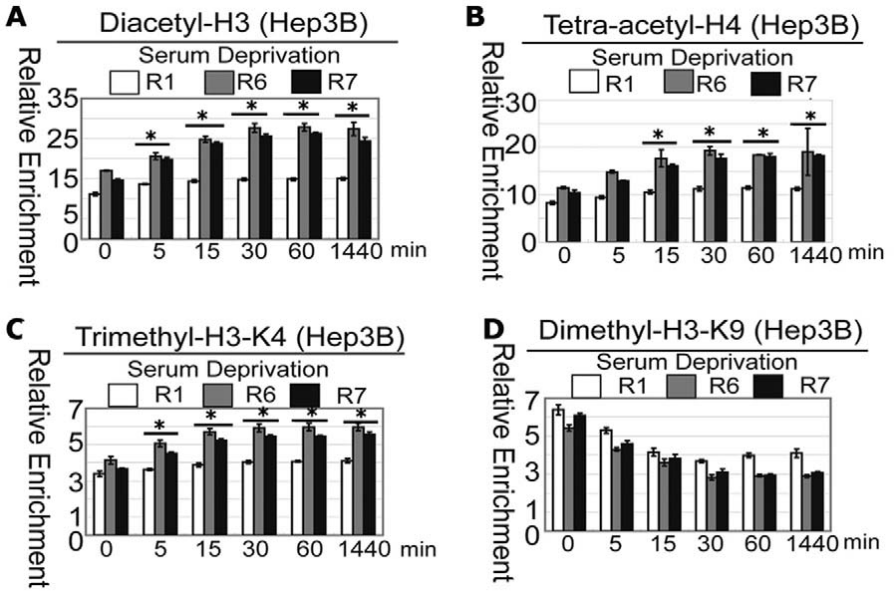
Serum deprivation induces changes in histone modification at the DRAM promoter locus in Hep3B cells. Hep3B cells were prepared for chromatin IP (ChIP) assays. The chromatin DNAs were immunoprecipitated with antibodies specific to H3K4me3 (A), anti-di-acetyl-H3 (B), anti-H3K9me2 (C) and anti-tetra-acetyl-H4 (D), and the enriched DNA fragments flanking the DRAM promoter were analyzed by quantitative PCR. Data were presented as the amount of DNA recovered by specific antibodies relative to DNA enriched by the appropriate IgG controls. The results were expressed as the means±standard deviations of three independent experiments. *: P<0.05.

To further strengthen the observation that the proximal region of the DRAM promoter contains putative cis-regulatory elements to serum deprivation, we examined the histone modification status at this locus using the ChIP assays. Histone acetylations such as lysine acetylation on H3 and H4 are generally known to be associated with actively transcribed genes and open chromatin structure [14]–[16]. Antibodies specific to diacetylated H3 (K9 and K14) and tetra-acetylated H4 (K5, K8, K12 and K16) were used to perform the ChIP analysis to determine the pattern of histone modifications at the DRAM promoter locus with or without serum. In Hep3B cells both H3 and H4 were acetylated at the R1, R6 and R7 region, with the R6 locus showing the highest acetylation level. The histone acetylation was apparently increased in a time-dependent manner upon serum deprivation at the R6 and R7 regions, but not the R1 region (Figure 4A, B)

Next, we examined the histone methylation status at the locus of the DRAM promoter. The trimethyl-H3K4, a euchromatic marker [17]–[18], displayed a similar pattern with Diacetyl-H3 and Tetra-acetyl-H4 in Hep3B cell (Figure 4C), while the dimethyl-H3K9 was scored lowest in R6 region at basal level and was decreased along with prolonged serum starvation (Figure 4D). The dimethyl-H3K9 serves as a signal for chromatin silencing by recruiting the HP1 proteins (heterochromatin protein 1) and is mutually exclusive with H3-K9 acetylation [19]. Taken together, these observations indicate that the transcriptional activation of the DRAM gene is correlated with increased local euchromatic markers and concomitantly decreased heterochromatic markers.

### NF-κB is not responsible for the activation of the DRAM promoter

**Figure 5:**
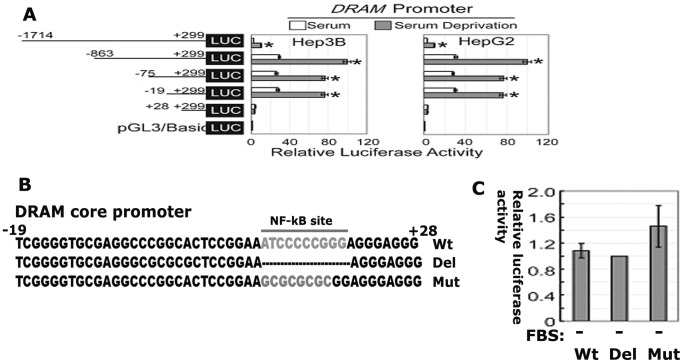
The putative cis regulatory elements responsive to serum deprivation is resided in the DRAM promoter region flanking −19/+28 nucleotides. (A) Hep3B and HepG2 cells were transiently transfected with reporter plasmids containing truncated versions of the promoter region of the *DRAM* gene as indicated. Luc-DCP is defined as the reporter containing the shortest promoter region (−19∼+28). (B) The core promoter region contains a putative NF-κB binding site. Transcription factor binding sites presented in the core promoter region (-19∼+28) were predicated by web software TFSEARCH and MatInspector and the mutation or deletion plasmids were generated using the site-directed mutagenesis method. (C) Mutation or deletion of the NF-κB binding site does not affect the core promoter activity.

To identify the minimal regulatory elements to serum deprivation in the DRAM promoter, various fragments of the proximal DRAM promoter were inserted into upstream of the firefly luciferase reporter vector. The transcriptional activities of these constructs were assayed in Hep3B and HepG2 cells. The reporter containing −1741/+299 fragment only weakly responded to serum deprivation, while the reporters containing −863, −75, 19/+299 fragments displayed significant high luciferase activities in responsive to serum deprivation. Strikingly, the reporter containing the +28/+299 fragment show no activity to serum deprivation (Figure 5A). These data indicate that the cis regulatory elements to serum deprivation in the DRAM promoter is resided the region flanking −19/+28 nucleotides (Figure 5B). The promoter reporter containing the shortest DNA fragment encompassing −19/+28 region was named as Luc-DCP hereafter.

In an effort to search for potential transcription factor binding sites in −19/+28 region, we next performed comprehensive bioinformatic analysis and found one canonical NF-κB-binding site resided in this region. To confirm if this putative NF-κB-binding site is functional, we made deletion and mutation in this site (Figure 5B). Surprisingly, deletion or mutation of this NF-κB-binding site had no apparent effect on the promoter activity in responsive to serum deprivation (Figure 5C). These data suggest that other factors, not NF-κB is required for DRAM induction by serum deprivation.

### Key components of the transcription machinery complex are bound to the proximal region of the DRAM promoter

**Figure 6:**
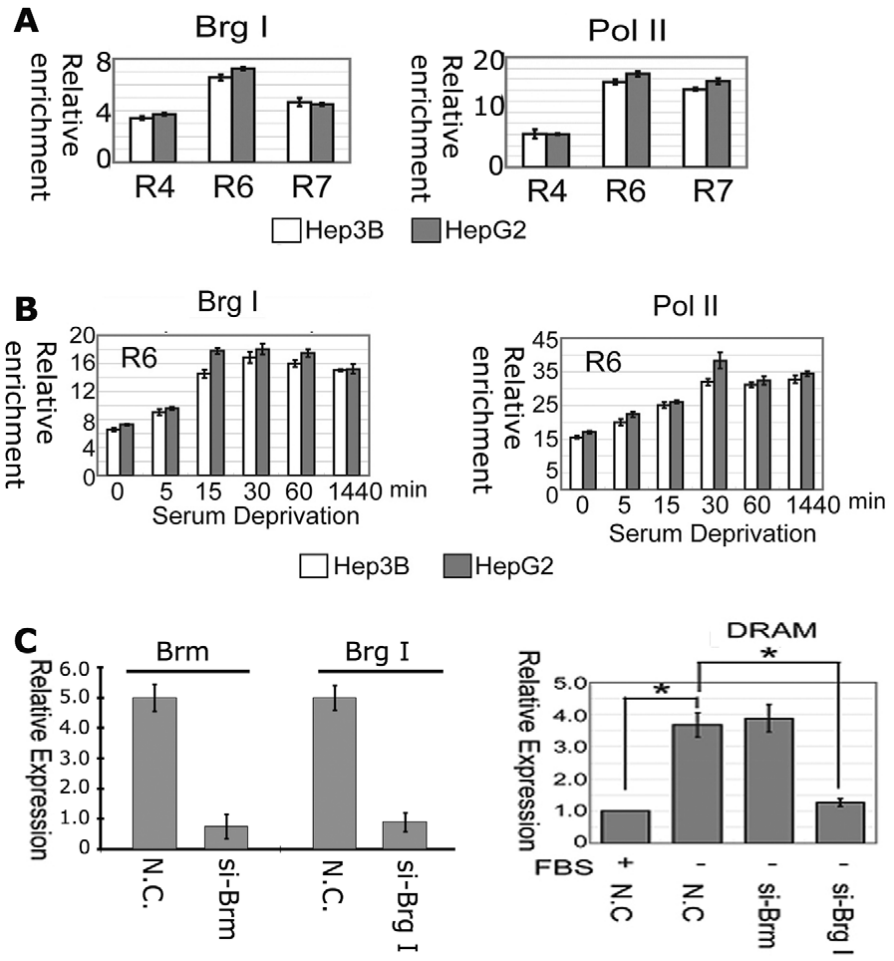
The chromatin remodeling factor Brg-1 is essential for serum starvation-induced DRAM expression. (A) Brg-1 is bound to the proximal region of the DRAM promoter. ChIP assays were performed in Hep3B and HepG2 cells. (B) Serum deprivation enhances Brg-1 and Pol II binding to the DRAM promoter locus. (C) Depletion of Brg-1 using specific targeting shRNA abolishes serum deprivation induced DRAM expression.

Brg I and RNA polymerase II are major components of RNA polymerase II holoenzymes which participate in gene transcription [20]. The chromatin from Hep3B and HepG2 cells were harvested and precipitated with anti-Brg I and anti-Pol II antibodies. The precipitated DNAs were evaluated by real-time PCR. Brg I bound the most to the DNA at the R6 region in both cells, while Pol II bound to the R6 and R7 regions with higher affinity than that of R4 region (Figure 6A). Strikingly, serum deprivation resulted in an increased binding of Brg I and Pol II to the DNA at the R6 region and such increases were seen as early as 5 min following the serum deprivation and reached the highest level at 30 min in both cells respectively (Figure 6B).

### Brg-1 is required for serum deprivation induction of DRAM expression

To examine the role of Brg I in induction of DRAM expression, HepG2 cells were transfected with siRNA oligos specifically against Brm or Brg I for 24 h followed by RT-PCR assays (Figure 6C, left panel). The induction of DRAM expression by serum deprivation was abolished by the depletion of Brg I. However, knockdown of Brm did not affect the expression of DRAM (Figure 6C). These results demonstrate that Brg-I is essential for DRAM induction in responsive to serum deprivation.

## Discussion

DRAM has a potential tumor-suppressive function and is critical for p53 to modulate autophagy and apoptosis [12]. Therefore, the discovery of regulatory factors that govern DRAM expression will provide targets for new therapeutics. However, the regulation of DRAM expression is poorly described so far. Here, we demonstrated for the first time that serum deprivation strongly induced DRAM expression in liver cancer HepG2 and Hep3B cells and identified a core DNA sequence in the DRAM promoter, which is essential for its responsiveness to serum deprivation.

Previously DRAM was identified as a p53 induced gene and a functional p53 response element is located at 2.3 kb upstream of the DRAM promoter [21]. Here, we demonstrated that serum starvation induces DRAM expression in HepG2, a p53 positive cell, and Hep3B, a p53 negative cells in a similar manner, suggesting p53 may not be a key factor involved in this induction [22]. However, it is a great interest to see if there is any cooperation between p53and serum deprivation in the regulation of DRAM expression.

Our data showed that the longer fragment (−1714) weakly responded to serum starvation. We postulate that in the longer fragment there exist repressive cis elements which could decrease the basal activity of DRAM promoter. The short version of the promoter does not contain such elements, and display high transcriptional activity.

Acetylation and methylation on lysine residues of the core histone tails are critical for gene transcription [23]–[24]. Acetylations of lysines on H3 and H4 are generally known to be associated with actively transcribed genes and open chromatin structure [15]–[16], [25]. Consistently, we observed that in Hep3B and HepG2 cells lysine acetylation on H3 and H4 was apparently increased in a time-dependent manner upon serum deprivation at the core promoter region of DRAM gene. The trimethyl-H3K4, a euchromatic marker for active transcription, displayed a similar pattern with Diacetyl-H3 and Tetra-acetyl-H4 in Hep3B cell. In contrast, the dimethyl-H3K9 was scored lowest in R6 region at basal level and was time-dependently decreased upon serum deprivation. The dimethyl-H3K9 serves as a signal for chromatin silencing by recruiting the HP1 proteins and is mutually exclusive with H3-K9 acetylation. These data suggest that serum deprivation may induce cooperative assembly of histone acetyltransferases, deacetylases and methyltransferases, transcriptional complex to the target chromatin to modify histone codes. However, we are not clear which histone modifiers are recruited to this locus. Interestingly, we observed that changes in repressive to active histone marks take place in first 15 minutes, and do not change significantly at all until 24 hrs, while the mRNA of DRAM were gradually increased and reached high level until at 24 hours and 48 hours. This apparent delay of DRAM mRNA expression may be due to the time for assembly of the transcription machinery to efficiently drive DRAM gene expression. However, the exact mechanism needs to be elucidated. Currently, we are attempting to identify the potential protein complexes which could bind to this core DNA sequence using DNA pulldown and Mass Spectrometry approaches and has identified multiple proteins including p300 which may potentially bind to the core promoter of DRAM. Their roles in the regulation of DRAM expression are now being thoroughly interrogated.

The tumor suppressor Brg-1 is a central component of the SWI/SNF chromatin- remodeling complex [26]. This complex can activate gene transcription by remodeling the chromatin structure upon disturbing DNA–histone interactions at the nucleosomes. We demonstrated that Brg-1 is enriched at the core promoter region of the DRAM gene and is required for serum deprivation induced DRAM expression.

The protein Brm (Brahma) can substitute Brg-1 and fulfill the helicase/ATPase function of the complex in vitro. However, Brg-1 and Brm are not functionally interchangeable in vivo. Homozygous *Brg1* knockout embryos die before implantation and heterozygous mice are prone to developing epithelial tumors [26]–[27], while *Brm* knockouts are viable and do not develop tumors [28]–[29]. Consistently, depletion of Brg-1 in HepG2 cells abolished serum deprivation induced DRAM expression, while depletion of Brm did not affect DRAM expression. Brg-1 has been shown to play key roles in the regulation of cell apoptosis and autophagy, but the underling mechanisms are obscure. Identification of DRAM is a direct target of Brg-1 will shed new light on the molecular regulation of autophagy.

## Materials and Methods

### Cell culture, transfections and plasmids

HepG2 and Hep3B cells (ATCC) were maintained in DMEM containing 10% FBS (Gibco), 2 mM L-glutamine, and penicillin (50 U/ml)/streptomycin (50 µg/ml) at 37°C under 5% CO_2_ in a humidified chamber.

Cells transfection was performed using the Lipofectamine 2000 (Invitrogen Corp, Carlsbad, CA, USA) according to the manufacturer's instructions. The siRNA oligos specifically targeting BrgI (CGCGCUACAACCAGAUGAATT), Brm (GGAUUGUAGAAGACAUCCATT) or negative control (scramble RNA, UUCUCCGAACGUGUCACGUTT) were purchased (GenePharma Co., Ltd, Shanghai, China).

### Reverse transcription PCR (RT–PCR)

Total RNA was extracted using the Trizol reagent (Invitrogen). The reaction mixture (20 µl) containing 1 µg of total RNA was reverse transcribed to cDNA in the usage of PrimeScript RT-polymerase (Takara). The resulting cDNAs were analyzed using quantitative PCR (Mx3000P real-ime PCR System, Stratagene, La Jolla, CA, USA) with primer pairs specific to DRAM and GAPDH. Data were presented as ratios of the total mRNA levels of DRAM against β-actin.

### 5′-RLM-RACE

The GeneRacer system (Invitrogen), based on RNA ligase-mediated and oligo-capping rapid amplification of cDNA, was carried out according to the manufacturer's instructions. The kit ensures the amplification of only full length transcripts by eliminating truncated messages from the amplification process. DRAM-specific primers (F, TGCGAAACGAGTGAAGTCACAAAGC; R, GCGAGCTCCAAGCGGACGCGACTAC) were used for amplification by PCR using the LaTaq GC-Rich PCR system (Takara, Inc., Dalian, China).

### Chromatin accessibility analysis

Accessibility of DNA to DNase I digestion and MNase (Takara, Inc., Dalian, China) were analyzed using chromatin accessibility through CHART-PCR as described previously [30]–[31]. The assays were repeated for three times independently. Primer pairs for different regions as followed: R1 (F: TCCAACCCTCATGGATGGCTTTTAG; R: ATTCAGTCACATCTTCAGGCTCTAC); R2 (F: ATGAATCAGTAGTAGGGCACGAAAG; R: CAGCCTGGTCAACAGATAGAG); R3 (F:TTGCGGGTCTCACTATATTGTCCAG, R: GGGCAGACTAGTATTTCAAGAGATC); R4 (F: TCCAGAACACAAATCCCTTTCACAG, R: GAGCCTTTATCACACCACTTCACTC); R5 (F: CGCTCTCCTGGAAAGCAGCACAATG, R: AGTGTTCAATGAGGTTCGCCAGGTC); R6 (F: ACCTCATTGAACACTTCTGCCCGAC; R: GCTCTTGCGCAACTCTGGAGAAAAG); R7 (F:CGAGTGTCCAAACCAAAAGCGAAAG; R: TGCGAAACGAGTGAAGTCACAAAG) .

### Chromatin immunoprecipitation (ChIP)

ChIP assays were performed according to the manufacturer's instructions (Active Motif, Carlsbad, CA, USA). The protein-DNA complexes were immunoprecipitated with 4 µg antibodies RNA polymerase II (SC-9001, Santa Cruz Biotechnology), and Brg1 (SC-10768); dimethyl-H3-K9 (ab1220, Abcam), tetra-acetyl-H4 (06-866, Upstate) and diacetyl-H3 (07-593, Upstate); and trimethyl-H3-K4 (9751, Cell Signaling Technology). Real-time PCR reactions were performed in triplicate with 1 µl of precipitated DNA. DNA recovered from samples containing an antibody was compared with IgG controls performed on aliquots from the same chromatin preparation. Data were presented as the amount of DNA recovered relative to the appropriate IgG control provided by the manufacturers. The results were expressed as the means±standard deviations of three independent experiments.

### Construction of the DRAM promoter-luciferase reporter plasmids and the luciferase assay

PCR assays were performed using primer sets specific to the *DRAM* promoter (F-1714): AGGAACGCGTACTCCACGGCCACAGTGATCTCTTG; F(−863): TGGAACGCGTGGCCACATATGTTGGGCTCAGACTC; F(−75): TGACACGCGTGGTGGCCACTCTCACTGCTGCGAAG; F(−19): TGACACGCGTTCGGGGTGCGAGGCCCGGCACTC; F(+28): TGACACGCGTGTTGGGAGAAATAAATCCTTTTCTC; R(+299): CGTACTCGAGCGGGCTTGTTGCGAAACGAGTGAAG). These PCR products were cloned into the Mlu I/Xho I sites of the pGL3-Basic vector (Promega, Madison, WI, USA). Promoter mutations and deletions were generated by a PCR-based site-directed mutagenesis kit (Takara), using corresponding plasmid as the templates. For the nucleotide substitution and deletion of the minimal promoter, an eight nucleotide DNA sequence GCGCGCGC was used to replace the wild type DNA in the minimal promoter, or the core NF-kB binding site was deleted using the site directed mutagenesis method. Promoter reporter plasmids were transiently transfected into cells using Lipofectamine 2000 (Invitrogen). pTRL-TK plasmid (Promega) was co-transfected as an internal control to evaluate transfection efficiency. Twenty-four hours posttransfection, the resulting cells were harvested and prepared for luciferase activity analysis. The luciferase activities were measured using the dual-Luciferase reporter assay system (Promega) according to the manufacturer's protocol with the illuminometer (PerkinElmer Life Sciences, Boston, MA, USA).

### Western Blot Analysis

Cells were harvested and lysed in RIPA buffer (Beyotime Inc.). The cell lysates were clarified by centrifugation at 15,000 g for 10 min. 20 µg of the total proteins was resolved on 8% SDS-PAGE gels. The proteins were electrophoretically transferred onto Immobilon P membrane (Millipore). The western blots were described previously [32]. The antibodies DRAM (AP1825a,Abgent) and β-actin (SC-130656, Santa Cruz Biotechnology) were purchased.

### Statistical analysis

Statistical evaluations were conducted using the t-test. P-values<0.05 were considered to be statistically significant.
