# Correction: Serum Starvation Induces DRAM Expression in Liver Cancer Cells via Histone Modifications within Its Promoter Locus

**DOI:** 10.1371/annotation/cd548ece-6f43-4837-9936-7f5d282c17b8

**Published:** 2013-06-19

**Authors:** Peihua Ni, Hong Xu, Changqiang Chen, Jiayi Wang, Xiangfan Liu, Yiqun Hu, Qishi Fan, Zhaoyuan Hou, Yang Lu

There was an error in affiliation 1 for authors Peihua Ni and Yang Lu. Affiliation 1 should be: Department of Pharmacy, Shanghai Jiaotong University, School of Medicine, Shanghai, China. 

